# SNPPicker: High quality tag SNP selection across multiple populations

**DOI:** 10.1186/1471-2105-12-129

**Published:** 2011-05-02

**Authors:** Hugues Sicotte, David N Rider, Gregory A Poland, Neelam Dhiman, Jean-Pierre A Kocher

**Affiliations:** 1 Department of Biomedical Statistics and Informatics, Mayo Clinic, 200 1st street SW, Rochester, MN 55905, USA; 2 Department of Internal Medicine, Mayo Clinic, 200 1st street SW, Rochester, MN 55905, USA

## Abstract

**Background:**

Linkage Disequilibrium (LD) bin-tagging algorithms identify a reduced set of tag SNPs that can capture the genetic variation in a population without genotyping every single SNP. However, existing tag SNP selection algorithms for designing custom genotyping panels do not take into account all platform dependent factors affecting the likelihood of a tag SNP to be successfully genotyped and many of the constraints that can be imposed by the user.

**Results:**

SNPPicker optimizes the selection of tag SNPs from common bin-tagging programs to design custom genotyping panels. The application uses a multi-step search strategy in combination with a statistical model to maximize the genotyping success of the selected tag SNPs. User preference toward functional SNPs can also be taken into account as secondary criteria. SNPPicker can also optimize tag SNP selection for a panel tagging multiple populations. SNPPicker can optimize custom genotyping panels including all the assay-specific constraints of Illumina's GoldenGate and Infinium assays.

**Conclusions:**

A new application has been developed to maximize the success of custom multi-population genotyping panels. SNPPicker also takes into account user constraints including options for controlling runtime. Perl Scripts, Java source code and executables are available under an open source license for download at http://mayoresearch.mayo.edu/mayo/research/biostat/software.cfm

## Background

Despite the commercial availability of affordable genome wide genotyping panels, custom-designed SNPs panels are frequently used for high resolution genotyping studies focusing on specific genes or chromosomal regions. The design of custom SNP panels for genotyping studies aims to minimize the number of SNPs to genotype while maximizing the information content of the panel.

The number of SNPs to genotype can be minimized by taking advantage of linkage disequilibrium (LD) between SNP alleles in the same population. A number of algorithms are available to assess LD between SNPs and select tag SNPs representative of groups of correlated SNPs called bins [1-5]. These bin-tagging algorithms use population specific sets of reference genotypes to compute bins and tag SNPs and report all possible tag SNP candidates for each bin. Note that Tagger, which reports the best tag SNP, also provides an exportable table of r^2 ^values between SNPs that can be used to compute all tag SNPs candidates. Since these tag SNPs candidates are theoretically equivalent from a linkage disequilibrium point of view, only one tag SNP per bin needs to be genotyped to account for the genetic variation of the SNPs in that bin. In practice, choosing tag SNPs based on LD criteria alone, without considering assay constraints, can lead to selecting tag SNPs that might fail experimentally. Therefore the design of a panel needs to account for experimental factors to maximize genotyping success. These factors include genotyping score (provided by the vendor) and distance constraint between SNPs. Furthermore, cost constraint should also be taken into account. For instance, when a SNP panel is designed to genotype two or more populations, SNPs that tag bins in multiple populations should be prioritized. Finally, user-defined preferences such as inclusion or exclusion of specific SNPs and their prioritization by functional category (e.g. non-synonymous) should also be accounted for in the design of the panel. Therefore the design of a SNP panel is a complex task that involves the optimization of multiple parameters. Applications like mPopTag[5], TagZilla[3], TAGster[6], multiPopTagSelect [7], and Snagger [8] provide partial solutions to this problem while also including some level of support for multi-population tag SNP selection.

However, none of these applications provides control over a comprehensive enough set of parameters to effectively customize and optimize tag SNP selection for the Illumina Infinium assay. SNPPicker was developed to fill this gap and since the Infinium assay can support custom panels with up to 200,000 SNPs, SNPPicker also support simultaneous tag SNP selection over multiple genes or chromosomal regions. SNPPicker is an application for the design of genotyping panels that accounts for experimental platform constraints, user specific preferences, and optimal selection of tag SNPs across multiple populations. SNPPicker focuses on the Illumina platforms since Affymetrix and Applied Biosystems (ABI) provide their own procedures for tag SNP selection. However, configuration parameters can be adjusted to apply to other platforms.

## Methods

SNPPicker is a post-processor of LD bin-tagging algorithms. The application can process the output of ldSelect, TagZilla, Tagger, and Snagger to obtain tag SNPs and bin definitions. Alternatively, bin definitions, number of SNPs per bin, and tag SNPs per bin can be specified via tab-delimited files. SNPPicker can be configured for different genotyping platforms and user preferences. A command line interface enables control of SNPPicker options and specification of project-specific input and output files.

### Optimization Constraints

The optimization process takes into account several factors such as:

### Platform-Specific Factors

*Conflicting tag SNPs: *SNPs that are in close proximity along the DNA sequence can interfere with each other when assayed [9]. For instance, Illumina recommends SNPs to be separated by more than 60 bp on their GoldenGate assay [10]. SNPPicker optimizes the avoidance of conflicting tag SNP based on a distance cut-off. In addition, the user can request the distribution of conflicting tag SNPs across multiple panels to force conflicting tag SNPs to be genotyped. When specified, this request will be first taken into account by SNPPicker. Remaining conflicts will be resolved by selecting non conflicting tag SNPs.

*Genotyping Probability: *the probability that a SNP will be successfully genotyped depends on several physico-chemical and experimental factors that can be empirically assessed. Ingersoll and co-workers suggested combining a predicted genotyping score provided by vendors and experimentally established confidence classes into a probability reflective of the chance that a SNP has to succeed during the assay [11,12]. SNPPicker uses a similar approach, allowing the specification of two properties per SNP that are converted into probabilities. By default Ingersoll et al. parameters for are loaded into SNPPicker. The mapping function is under user control, but SNP probabilities must be limited to a small set of discrete values to enable functional prioritization.

*Illumina Infinium-specific factors: *the Infinium assay developed by Illumina introduces a new design constraint. SNPs with rare complementary allele combinations (i.e. A/T or C/G) are genotyped using pair of bead types whereas all other allele combinations are genotyped with only a single bead type [10]. Since the panel include a fixed number of bead types, the total number of SNPs that can be included in a panel can be maximized by avoiding A/T or C/G SNPs. SNPPicker can be used to perform this optimization.

### User-Specified Factors

*Functional rank: *different functional categories can be assigned to a tag SNP such as nonsense, missense, non-synonymous, coding, etc. Each category is assigned a configurable rank to define the order of preference in which tag SNPs will be included in the assay panel. This rank prioritization is only taken into account when tag SNPs have the same genotyping probability.

*Number of assay panels: *SNPPicker can organize SNPs on multiple SNP panels. When more than one panel is used, SNPPicker attempts to remove conflicting tag SNPs by distributing conflicting pairs across panels. Conflicts due to DNA template competition can thus be avoided.

*Maximum number of tag SNPs per bin: *the maximum number of tag SNPs to select per bin can be defined by the user. This number can be assigned as a function of the total number of tag SNPs. The default is one tag SNP per bin. This feature is useful to avoid failures for large bins which represent a large fraction of the genetic variation.

*Maximum genotyping probability of a bin: *a genotyping probability threshold can be set in the program. This threshold defines the limits above which the user considers that a bin will be successfully assayed. From a technical point of view, this threshold speeds up the search for solutions since once the threshold is reached tag SNPs are no longer added to a bin even if the maximum number of tag SNPs per bin has not been reached. By default the threshold value is set to 1, which turns off this optimization since no bin can reach that probability.

*Existing genotypes: *SNPPicker can account for SNPs that have been genotyped in a previous experiment. SNPPicker will not pick any tag SNPs for bins where these genotyped SNPs are tag SNPs.

*Minimum genotyping score threshold for a SNP: *a threshold can be set to exclude SNPs with a low predicted genotyping score from the optimization process. In addition to having a higher risk of failure, it is suspected that some of these SNPs can interfere with the performance of other SNPs in the panel by non-specifically binding to DNA[9].

*User imposed tag SNPs inclusion and exclusion: *the user can force tag SNPs to be present (also called obligates) or excluded from the final assay panel. If two obligate SNPs are in violation of the proximity constraint, the one being selected will be decided based on the optimization of the score of each alternative.

### Optimization Strategy: Bin Clusters

SNPPicker's optimization strategy focuses on finding a solution that maximizes the score of a panel while minimizing the total number of tag SNPs or bead types to genotype. Since the exhaustive enumeration of all possible tag SNPs panels that match user defined criteria can be time consuming, SNPPicker groups non-independent bins into clusters for joint optimization. Non-independent bins are transitively grouped by single linkage clustering. Two bins are non-independent when each has a member of a pair of conflicting tag SNPs or when these bins share at least one tag SNP (which can occur when bins are from different populations). Each resulting cluster of bins, called a bin cluster, is optimized and scored independently during the optimization process.

The tag SNPs selected from each bin cluster are combined into the final panel and scored as a function of the genotyping probability of the SNPs in the panel. Optimization first maximizes coverage criteria and then a score for each bin cluster. Lastly, the sum of the functional classes of all the selected tag SNPs is minimized for solutions with equal bin cluster score.

### Scoring Functions

#### Coverage Selection Criteria

The panel has to optimize the following coverage criteria for each bin cluster before the bin cluster score is optimized.

(1) Has the most number of bins tagged by at least one tag SNP.

(2) Has the maximum number of obligates.

(3) Has the lowest difference between the expected and actual total number of tag SNPs in the panel. The expected total number of tag SNPs is derived from the maximum number of tag SNPs per bin set by the user. No penalty is applied if a bin has less than the user defined number of tag SNPs because the bin has too few tag SNPs or because the bin probability reaches the user defined maximum genotyping probability of a bin.

#### Bin Cluster Score

The bin cluster score is computed from the genotyping probability of a bin divided by the total number of tag SNPs to favor a solution with fewer tag SNPs. The ratio is weighted by the number of tag SNPs in a bin, a strategy [2] that increases the statistical power for association with a phenotype.

The score of *S_P _*a panel *P *is computed by summing scores over each bin cluster:

where the score for the *k^th ^*bin cluster is computed as follows:(1)

where *P_i_^tagged_bin ^*is the probability of successfully genotyping at least one tag SNP per bin, *n_i_^tagSnps ^*is the number of tag SNP in *bin i*, and *n*_*k*_^*tag*s ^is the total number of tag SNPs selected for bin cluster *k*. Note that if the -infinium command line option is selected, *n*_*k*_^*tag*s ^in equation 1 is replaced by the number of bead types *n*_*k*_^*beads*^.

The probability *P*_*i*_^*tagged_bin *^is computed from

where the probability *P_l_^snp_success ^*of successfully genotyping a SNP is a configurable function of the predicted genotyping score and the confidence class. *P_l_^snp_success ^*is obtained from retrospective analysis of SNPs that have been successfully genotyped.

#### Functional Prioritization and Functional Score

During the first two phases of solution search, tag SNPs with equal probability are considered for inclusion in the panel in order of their functional rank (higher rank first) insuring that the first solution found with a given coverage and score will have the best functional rank among equivalent solution. However, during the search for a final solution, a swapping procedure is used (section 2.4), which breaks the functional ordering. During that phase, the functional rank prioritization is achieved by picking the solution with the maximum sum of the functional rank of the tag SNPs in the bin-cluster (after coverage and bin cluster score optimization).

### Optimization Algorithm

#### Pre-processing

Prior to optimization, tag SNPs are organized to facilitate and speed up the enumeration of solutions. Three pre-processing steps are executed in the following order:

(1) Selection of initial set of candidate tag SNPs: during this step, tag SNPs not meeting minimum score criteria and SNPs excluded at the user's request are removed from the list of tag SNPs.

(2) Identification of bin clusters: next, bin clusters are created by linking bins with conflicting tag SNPs or sharing overlapping tag SNPs. The latter happens when multiple populations are analyzed. Note that remaining isolated bins are treated as bin clusters.

(3) Ranking of tag SNPs in bin cluster: lastly, tag SNPs in each bin cluster are sorted in descending order of genotyping probability. Since tag SNP probabilities are discretized, many tag SNPs may have the same probability. Tag SNPs with the same probability are ordered by decreasing functional rank.

#### Optimization Procedure

SNPPicker's optimization procedure operates on each bin cluster of tag SNPs independently and proceeds in three consecutive phases. The first phase is designed to ensure rapid convergence towards a nearly optimal solution via a heuristic algorithm. The second and third phases further explore the solution space until the user specified time limit is reached or an exhaustive search has been completed.

##### Phase 1: nearly optimal solution

The first phase focuses only on non conflicting tag SNPs and tag SNPs genotyped by a single bead when the Illumina Infinium protocol is used.

*Single population optimization*: for each cluster, tag SNPs are selected for inclusion in the panel in order of ranking until the maximum genotyping probability threshold or the maximum number of tag SNPs has been reached for each bin in the cluster.

*Multi-population optimization*: a nearly optimal solution is created using a dynamic programming search: tag SNPs are ordered in each cluster as a function of the number of populations they tag (NPtag), and their rank in the bin cluster. Each tag SNP with the same NPtag is selected iteratively. Once a tag SNP is selected, NPtag is updated for the remaining tag SNPs, counting only bins that still need more tag SNPs. The remaining tag SNPs are then reordered as a function of NPtag and their rank in the list. When only tag SNPs tagging single bins remain, tag SNPs are picked independently for each bin. This procedure is repeated recursively until the maximum bin cluster probability score or the maximum number of tag SNPs has been reached for each bin in the cluster. The best solution serves as the starting point for phase 2.

##### Phase 2: swapping in conflicting tag SNPs and tag SNPs assayed by a pair of bead types

The second step is more time consuming. It attempts to add to the panel tag SNPs from pairs of conflicting tag SNPs or tag SNPs requiring a pair of bead types when the -infinium option is specified. A swapping strategy similar to the one described by Howie and co-workers is used [7] by simultaneously adding one or more of the not previously considered tag SNPs while removing subsets of tag SNPs from the panel to avoid proximity conflicts or superfluous coverage.

##### Phase 3: exploring full solution space

Finally in an attempt to further refine the nearly optimal solution obtained from previous phases, the systematic replacement of zero or more selected tag SNPs already in the panel with the remaining tag SNPs is performed. Since this swapping procedure can be time consuming, a time limit can be set by the user. Tag SNP swapping is performed independently for each bin cluster. Note that the number of removed tag SNPs can be different from the number of added tag SNPs to ensure the exhaustive exploration of solutions that meet the coverage requirements, yet have a better score. The swapping process stops when the time limit allocated for the search is exhausted or the full search for a better solution is complete. If a better solution is found by swapping, the entire swapping procedure is repeated with the new baseline solution. The numbers of SNPs to swap out is limited to be no more than 20 to limit computational time.

### Approximate solution

Bin clusters with a large number of tag SNPs can require more time to optimize than the limit set by the user. When the time limit is reached or if the cluster has more than 31 tag SNPs to swap, the exhaustive search from Phase 3 is reduced to a simpler version that sequentially tries to swap in a single tag SNP at a time instead of multiple tag SNP while still trying to swap out multiple tag SNPs. The tag SNPs are considered in the order of the ranked tag SNP list. Although not optimal, the time to find a high scoring solution is significantly reduced by avoiding swapping in multiple SNPs at the same time.

### Final Panel and report

The tag SNP panel is reported in a tabular format: obligate tag SNPs are listed first followed by the remaining tag SNPs. The predicted genotyping score, functional class, bin ID, population tagged, and genotyping probability is also provided for each SNP. Other than obligates, tag SNPs are reported in order of their contribution to the panel score. The incremental contribution to the panel score of each tag SNP is listed as well. If the multiple panel design option was selected, a panel identifier is provided for the conflicting tag SNPs. Finally, if a bin has no selected tag SNP, the report includes the reason why each tag SNP in that bin was not selected.

### Integration of SNPPicker with SNPApp

SNPApp is an in-house application developed to facilitate the selection of SNPs and computation of tag SNPs in genes or chromosomal regions. SNPApp accesses multiple sources of public reference genotypes including Hapmap[13] Phase II, release 23, NIEHS SNPs[14], and SeattleSNPs[15]. The application has recently added genotypes from the 1000 genomes project[16]. Gene definitions are obtained from Entrez RefSeq[17-20]. SNPApp uses ldSelect for tag SNP calling[1]. SNPApp returns LD bins and tag SNP data for each gene or chromosomal region submitted as input. SNPApp provides this information for each source of reference genotype along with coverage information. SNPPicker is interfaced to SNPApp to provide a comprehensive solution for genotyping panel design. For this work, SNPApp was used to generate tag SNPs with the following parameters: r^2^>= 0.9, minor allele frequency cut-off of >= 5 percent, and inclusion of SNPs up to 10 Kb 5' or 3' of each gene.

## Results

### Validation Datasets

#### 55 Genes validation set

SNPPicker was used to design a 55 gene single-population GoldenGate panel for the Hapmap CEU population. The 55 innate and adaptive immune response genes dataset was assembled from 3 classes of genes. The first class includes innate immune response genes including antiviral proteins and associated pathway genes, interferon and interferon inducible genes, toll-like receptor and associated pathway genes (MX1, MX2, OAS1, OAS2, OAS3, ADAR, EIF2AK2, IRF3, IRF7, ISG20, ISGF3G, RNASEL, DDX58, VISA, CASP10, TRIM22, TLR3, and TLR4). The second class includes a broad spectrum of immune response genes such as cytokine (IL2, IL4, IL5, IL6, IL10, IL12A, IL12B, IFNA1, IFNA2, IFNA21, IFNB1, IFNG, TNFA, and CSF2) and cytokine receptor genes (IL2RA, IL2RB, IL2RG, IL4R, IL6R, IL6ST, IL10RA, IL10RB, IL12RB1, IL12RB2, IL18R1, IFNAR1, IFNAR2, IFNGR1, IFNGR2, TNFRSF1A, TNFRSF1B, and CSF2RB) regulating Th1, Th2, and inflammatory responses to rubella. The third class includes genes encoding nuclear receptors for vitamin A and D (RXRA, RARA, RARB, RARG, and VDR) that play an important role in the regulation of both innate and adaptive responses to viral vaccines.

A total of 1995 tag SNPs were provided to SNPPicker using SNPApp, with 1790 being above the minimum score threshold. 130 of those tag SNPs being considered for the panel (7.1%) had at least one other tag SNP in close proximity (closer than 61 bp).

#### 160 Genes validation set

SNPPicker was also used to design a multi-population GoldenGate panel to genotype 160 cardiovascular disease related genes [21,22]. Tag SNPs were extracted from Hapmap for the European whites (CEU) and African Yorubans (YRI) population using SNPApp. The analysis of the 9135 tag provided to SNPPicker showed that 3873 tag SNPs (42.2%) are shared between the CEU and YRI population. 1948 tag SNPs (21.3%) have at least one other tag SNP in close proximity (closer than 61 bp).

### Validation

#### Quality of the optimization procedure

SNPPicker was configured to generate a single panel optimized for the Golden-Gate assay with a single tag SNP per bin. Pairs of tag SNPs separated by 60 bp or less were not allowed in the final panel. Tag SNP selection was performed independently for each chromosome containing at least one gene, allowing up to 50 seconds of optimization per bin cluster. For the 160 and the 55 gene panels, out of a total of 4263 and 1876 bin clusters optimized respectively, 7 and 2 bin clusters respectively were not completed within the fifty seconds allocated due to too many tag SNPs. The approximate solution was returned for those clusters. The computational time was dominated by the time spent to optimize the large bin-clusters.

#### Quality of the designed panel

To assess the quality of the solution returned by SNPPicker, one million random solutions per chromosome were generated for each panel. These solutions were constrained to include as many or fewer tag SNPs than the optimized panel returned by SNPPicker. Because of conflicts, some bins may not end up with any selected tag SNP, therefore bins need to be assigned tag SNPs in randomized order to avoid bias about which bins do not get tagged. For each chromosome, random solutions were generated by iteratively randomly selecting a bin and randomly assigning a tag SNP to that bin. The procedure for each solution terminates when i) the total number of tag SNPs in the panel has been reached or ii) no more tag SNPs can be added to any bin because of conflicts or because all bins have reached their maximum number of tag SNPs. This randomization procedure takes into account the selection of multiple SNPs per bin.

None of the random solutions had better coverage than SNPPicker's solution. For the random solutions with equal coverage, none of the random solutions for any of the chromosomes had scores better than SNPPicker's optimized solution. The results were similar for the 55 genes panel. The same analysis was also performed independently on each of the bin clusters for which an approximate solution was generated. In these cases as well, after one million permutations for each of those bin clusters, no better solutions were found.

#### Enrichment of high probability tag SNPs

**Figure 1:**
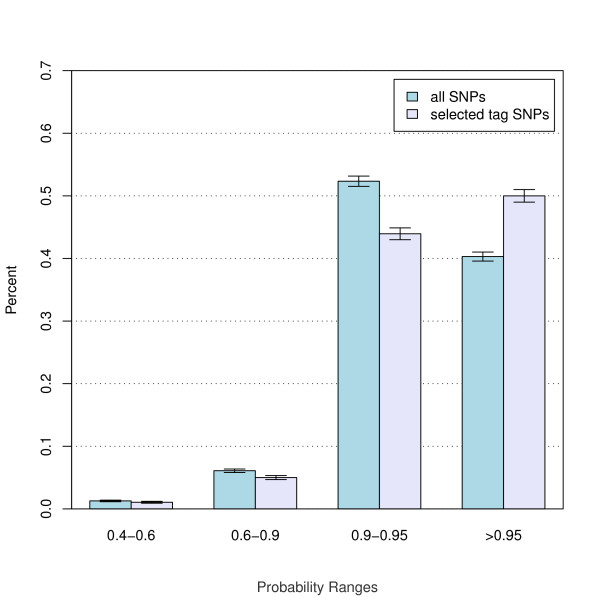
**Tag SNPs per Probability Range**. Tag SNPs genotyping probabilities before and after optimization. Only tag SNPs meeting the minimum score for inclusion in the panel are included in the histogram. Counts per bin are below each histogram bin and error bars at the top of bins are poisson estimates (the square root of the counts) scaled to percentage.

To assess the enrichment of high probability tag SNPs upon optimization, the normalized distribution of tag SNP probabilities in the initial set of tag SNPs was compared to that of the panel after optimization (Figure 1). The highest enrichment of 25% (from 40% to 50%) is observed for tag SNPs with probability>0.95. The probability enrichment is limited because the optimization can only operate on bins that have more than a single tag SNP (29% (2065/7295) of bins).

#### Enrichment of functional tag SNPs

**Figure 2:**
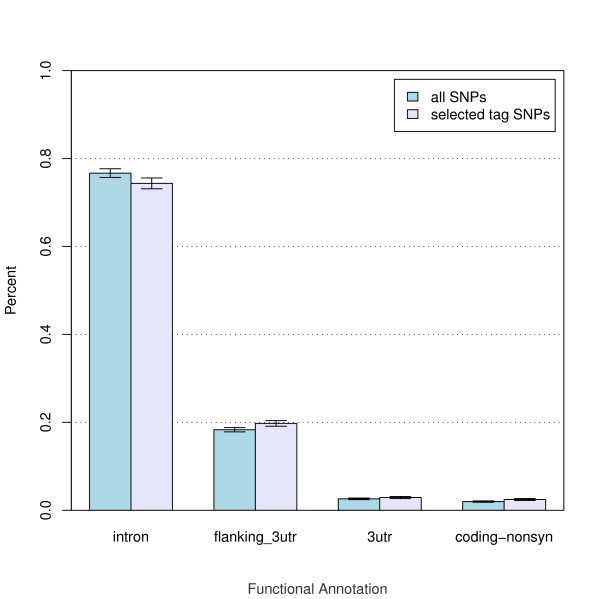
**Tag SNPs Functional Enrichment**. Tag SNPs functional class enrichment before and after optimization. Each bin represents a single rank of functional class (most significant classes on the right). Only ranks with at least 100 tag SNPs prior to optimization are shown. Only tag SNPs meeting the minimum score requirements for inclusion in the panel are included in the histogram. Counts per bin are below each histogram bin and error bars at the top of bins are poisson estimates (the square root of the counts) scaled to percentage. Multiple functional classes match each rank, only one label shown for each rank.

Figure 2 shows the enrichment of preferred functional classes in the designed panel, with the highest priority classes to the right of the figure. Contrary to the genotyping probability optimization, enrichment of functional classes is very limited. This is due to the small number of bin with multiple tag SNPs having identical genotyping probabilities. The lowest priority class is decreased by 2.5% upon optimization while the higher priority classes show slight increases.

#### Feature comparison with other tag SNPs selection applications

Table 1 compares the features of SNPPicker with two of the most commonly used applications for tag SNP selection with multi-population support: multiPopTagSelect V1.1 [7] and Snagger [8]. MultiPopTagSelect, like SNPPicker, post-processes the output of tag SNP selection algorithms. MultiPopTagSelect is designed to post-process the output of ldSelect only and optimize tag SNPs across multiple populations. MultiPopTagSelect takes into account scores assigned to SNPs and a functional rank of tag SNPs. In contrast with SNPPicker, multiPopTagSelect chooses functional rank over score and does not account for conflicting tag SNPs. Snagger is an application designed for single gene/region analysis that can design panels for multiple populations, but unlike SNPPicker, it uses a non-optimal incremental approach to select tag SNPs in multiple populations. It first optimizes tag SNPs selection for one population and next extends the selection to another population by adding SNPs to ensure coverage. This selection strategy was shown to be suboptimal [7]. SNPPicker is also the only tool whose scoring function gives different probability to tag SNPs with experimental validation or that can optimize panel design for the Illumina Infinium chemistry.

**Table 1:** Differentiating features between various multi-population tag SNPs selection programs

SNPPicker	Snagger	multiPopTagSelect	Feature
X		X	Simultaneous multi-population optimization

X	X		Optimizes genotyping score

X	X		Optimizes conflicting tag SNPs

X	X	X	Functional class prioritization (strategy specific to each application)

X	X		Optional selection of multiple tag SNPs per bin

X			Simultaneous optimization of multiple genes or regions

X			Accounts for previously genotyped SNPs

X			Optimizes for the Infinium assay

X			Distribute conflicting SNP across multiple panel

X		X	Not limited to Hapmap Samples

Differentiating features between the multi-population tag SNPs algorithms. Features similar to all 3 applications were omitted.

#### Optimization Comparison with Snagger and MultiPopTagSelect

The performance of SNPPicker, Snagger, and MultiPopTagSelect was compared on the design a SNP panel for the genotyping of the 160 genes validation set. For this comparison, two panels were designed for Infinium and GoldenGate assays. To make results comparable, the same tag SNP and LD information produced by Snagger was converted to bins in order to perform the comparison between the three applications. Tag SNPs were computed to tag SNPs with minor allele frequency greater of equal to 0.05 at a minimum r^2 ^of 0.9. Bins were created according the procedure in [1], but limiting the central tag SNPs to the tag SNPs selected by Snagger and proceeding in the population order of Snagger (CEU first, next YRI). It should be noted that the bin construction procedure led to different number of bins in the two panels because Snagger chose different tag SNPs in the two cases.

Snagger was run with the -minBinSize option set to 10000 and minProbSucc to 0.01 to disable the selection of multiple tag SNPs per bin. Genotyping scores were obtained from Illumina. Missing scores were set to -99. A minimum genotyping score of 0.4 was required for a SNP to be selected as candidate tag SNP. SNPPicker was limited to using at most 50 seconds per bin cluster to make the total runtime of all applications similar.

Tables 2 & 3 provide an overview of the multi-population panels designed by SNPPicker, multiPopTagSelect, and Snagger. Table 2 reports the comparative statistics of the panel designed for the GoldenGate assay that is sensitive to SNPs in close proximity (conflicting SNPs). Note that multiPopTagSelect does not handle conflicting tag SNPs. From the 7996 bins produced by Snagger, 7648 are tagged by SNPPicker compared to 7859 by multiPopTagSelect. However, multiPopTagSelect includes in the panel 426 conflicting SNP that might lead to assay failure, resulting in only 7433 bins that are likely to be successfully tagged. When compared to Snagger, the other tool that can handle conflicting SNPs, SNPPicker is able to tag 8 additional bins using only 5038 tag SNPs instead of the 5154 tag SNPS required by Snagger. SNPPicker can also design a panel that tags all of the 7859 bins tagged by multiPopTagSelect by spreading the conflicting tag SNPs across multiple panels.

**Table 2:** Multi-population panel design for the GoldenGate assay

optimizer	MultipopTagSelect	SNPPicker	Snagger
bins	7996	7996	7996
tagged bins	7859	7648	7640
tag SNPs	5248	5038	5154
conflicting tag SNPs	426	0	0

Table 3 reports comparative statistics on the panel designed for the Infinium assay. This assay does not require elimination of conflicting tag SNPs, but requires using two bead types for A/T or C/G tag SNPs versus a single bead type for all other SNPs. The cost of the panel, that is related the total number of bead types, is impacted by the number of A/T or C/G tag SNPs selected. SNPPicker is the only application able to optimize a genotyping panel to reduce the number of A/T or C/G tag SNPs to genotype while still maximizing the genotyping score (and functional score). The panel designed by SNPPicker includes 105 less bead types than MultiPopTagSelect and 195 less than Snagger. The difference between the number of tag SNPs selected by SNPPicker and MultiPopTagSelect comes from the optimization strategy used by SNPPicker. SNPPicker processes all loci on the same chromosome simultaneously, enabling saving 29 tag SNPs located into overlapping bins. These tag SNPs tagged genes within 20 KB of each other.

**Table 3:** Multi-population panel design for the Infinium assay

optimizer	MultipopTagSelect	SNPPicker	Snagger
bins	8023	8023	8023
tagged bins	7887	7887	7887
tag SNPs	5239	5210	5352
A/T or C/G tag SNPs	642	566	619
Total number of bead types	5881	5776	5971

## Discussion

SNPPicker automates the design of tag SNP genotyping panels with maximum likelihood of genotyping success while minimizing the number of tag SNPs to assay.

SNPPicker also optimizes functional tag SNPs, but only after maximizing genotyping probability. This approach makes SNPPicker different from other applications, such as multiPopTagSelect and Snagger, that prioritize functional class at the expense of the genotyping success of a SNP.

SNPPicker maximizes genotyping success by optimizing two properties: the genotyping probability of a bin (or a cluster of bins), statistically derived from the individual genotyping probability of each SNP; and, for some platforms, the proximity distance between SNPs. The genotyping probabilities currently used by SNPPicker are derived from a retrospective analysis of experimental genotyping results. SNP proximity is a strictly enforced constraint. Although, in this article, this feature was used only on the GoldenGate custom genotyping design, this constraint can significantly impact the design of the panel for any genotyping platform that is based on hybridization[9]. The importance of this effect was measured in two datasets. Fifteen and thirty three percent respectively of SNPs with minor allele frequency (MAF) >= 5% were within 60 base pairs of another SNP in the American European whites (CEU) population for the Hapmap II [13] (build 36, Feb. 21 2009) and for the 1000 genomes pilot 2 data (March 2010 release)[16]. SNPPicker also includes options to avoid any conflict, not only between selected tag SNPs, but between any SNP supplied by the user in the score files.

SNPPicker uses a time-constrained algorithm to search for an optimal solution. This option provides more flexibility and control over the time that will be allocated for the optimization, particularly when bin clusters with a large number of tag SNPs and spanning several populations have to be processed. The time allocated for the search is guided by the user. If the search completes before the time limit, the returned solution is optimal. If not, SNPPicker returns a solution that, while not guaranteed to be optimal, may in fact be the optimal solution.

Finally, SNPPicker includes a set of useful features that makes the tool versatile and easy to customize for the needs of a specific study. These features include: i) accounting for constraints of the GoldenGate or Infinium chemistry, ii) accounting for tag SNPs that have been previously genotyped (and therefore should not be re-assayed), iii) distributing tag SNPs on multiple panels to avoid proximity constraints, iv) simultaneous design of a multi-gene panel, and v) simultaneous multi-population optimization.

One limitation of SNPPicker's post-processing design is that if a bin has all of its tag SNPs excluded because of score or proximity constraint, the non-tag SNPs in the bin remain untagged. This limitation is easily mitigated by running a second round of tag SNP selection, only including SNPs that are in untagged bins and not in proximity conflicts with the tag SNPs chosen in the first pass.

## Conclusions

SNPPicker is an application for the design of single and multi-population genotyping panels based on the linkage disequilibrium of SNPs and additional constraints imposed by the user or by the genotyping assay. SNPPicker is currently the only tool that can optimize bead type selection for the Infinium assay, an assay that is frequently used when large SNPs panels have to be designed. Its integration with SNPApp provides an easy to use comprehensive solution to the design of genotyping panels. The extensive set of criteria that are offered to control the selection of SNPs make it also a flexible tool for the designed of customized SNP panels. Finally, SNPPicker is a command line application that can straightforwardly be integrated within data processing pipelines.

## Authors' contributions

HS and JPK equally contributed to the design of the method, the design of the experiments, and the manuscript preparation. HS wrote most computer codes and analyses. DR contributed the initial requirements and testing of the implementation. GAP and DNR contributed to the design of the 55 gene panel and edited the manuscript. All authors reviewed and approved the final manuscript.
